# Nobel Prize for physiology or medicine in 2023: how to dupe the cellular innate immune system using modified RNA for therapeutic treatment

**DOI:** 10.1007/s00424-023-02895-9

**Published:** 2023-12-13

**Authors:** Jan Medenbach, Herbert Tschochner

**Affiliations:** 1 Universität Regensburg, Regensburg Center of Biochemistry (RCB), 93053 Regensburg, Germany; 2 Universität Regensburg, Regensburg Center of Biochemistry (RCB), Biochemistry III, 93053 Regensburg, Germany

The 2023 Nobel prize in physiology or medicine was awarded to Katalin Karikó and Drew Weissman for their breakthrough discoveries to use modified RNA for therapeutic treatments. This enabled the development of life-saving COVID-19 RNA vaccines by the companies Pfizer-BioNTech and Moderna.

RNA is an extremely versatile molecule: it can e.g. store genetic information, serve as a structural component, and catalyze chemical reactions. This has fueled the hypothesis that it preceded DNA and protein in early evolution, giving rise to what is known as the RNA world. In all eukaryotic organisms including men, the genetic information is largely stored in the form of DNA and proteins catalyze most chemical reactions. However, RNA still plays a central role in gene expression and its regulation. It not only acts as the messenger molecule, providing the information for the synthesis of proteins, but it also delivers the necessary building blocks as aminoacyl-coupled tRNAs to the ribosome, which itself is a macromolecular machine that is largely comprised of RNA that also provides the catalytic activity for protein synthesis. Furthermore, noncoding RNAs are involved in regulation of many cellular processes including transcription, translation, RNA degradation or trafficking which underlines the general importance of RNA for each cell.

Given its essential role in gene expression, it is not surprising that RNA has been recognized as both a target, as well as an agent for therapeutic treatment. One therapeutic approach is to produce synthetic messenger RNAs (mRNAs) that serve as a template to program the expression of proteins in human cells. mRNAs are typically short-lived, and their encoded information does not become genetically fixed in the cellular genome, preventing its propagation to future cell generations. This allows transient, mRNA-directed production of proteins which is ideal for vaccination, a concept that was proposed more than 30 years ago [4, 6].

However, in vitro synthesized, therapeutic RNAs do not carry the RNA-modification imprint of a human cell. Therefore, they have a strong immunogenic potential which has for a long time diminished their efficiency and effectiveness in treatments [5]. The innate immune system of a human cell can discriminate between its own transcripts (‘self) and foreign nucleic acids (‘non-self) which are often encountered during bacterial or viral infection. The foreign nucleic acids differ slightly from the cellular ones, allowing their recognition by numerous pattern recognition receptors (e.g. Toll-like receptors) and cytoplasmic nucleic acid binding proteins that then trigger a potent interferon response or that eliminate the cell via apoptosis [8]. For effective vaccination with RNA, this cellular response needs to be, at least partially, suppressed.

Features of eukaryotic mRNAs such as the cap structure at the 5’ end and the 3’ poly-A tail improve stability of artificially synthesized mRNAs and increase protein expression from them. However, both modifications are insufficient to fully suppress immunogenicity of the RNA. In 2005, experimenting with dendritic cells, Katalin Karikó and Drew Weissman discovered, that mRNA base modifications that were introduced into the mRNA during in vitro synthesis, abrogate the cytokine response. Base methylation (m5C, m6A, m5U), thiouridylation (s2U) or pseudouridylation inhibit RNA recognition by Toll-like receptors and reduce cytokine release [2]. Incorporation of pseudouridine, an isomer of uridine, into mRNA turned out to be sufficient both to reduce immunogenicity and to improve translational capacity [3] which has subsequently allowed the development of an efficient vaccine against Zika virus infection [7].

The groundbreaking discoveries by Karikó and Weissman were also fundamental for the development of efficient mRNA-based vaccines to combat the SARS-CoV2 pandemic. Their rapid development and adaption to newly emerging virus strains, and their efficiency and effectiveness have not only saved the lives of many thousands of people, but also mark the beginning of a new era in vaccine development and production [1].

## Data Availability

No datasets were generated or analysed during the current study.
